# Investigation of the Structure and Corrosion Resistance of Novel High-Entropy Alloys for Potential Biomedical Applications

**DOI:** 10.3390/ma15113938

**Published:** 2022-05-31

**Authors:** Marzena Tokarewicz, Małgorzata Grądzka-Dahlke, Katarzyna Rećko, Magdalena Łępicka, Kamila Czajkowska

**Affiliations:** 1 Department of Materials and Production Engineering, Faculty of Mechanical Engineering, Bialystok University of Technology, ul. Wiejska 45 C, 15-351 Bialystok, Poland; m.dahlke@pb.edu.pl (M.G.-D.); m.lepicka@pb.edu.pl (M.Ł.); 75726@student.pb.edu.pl (K.C.); 2 Faculty of Physics, University of Bialystok, K. Ciołkowskiego 1L, 15-245 Bialystok, Poland; k.recko@uwb.edu.pl

**Keywords:** high-entropy alloys, corrosion properties, Al_0.7_CoCrFeNi, CoCrFeNiCu, TiAlFeCoNi, Mn_0.5_TiCuAlCr

## Abstract

High-entropy alloys are a new generation of materials that have attracted the interest of numerous scientists because of their unusual properties. It seems interesting to use these alloys in biomedical applications. However, for this purpose, the basic condition of corrosion resistance must be fulfilled. In this article, selected corrosion properties of self-composed high-entropy alloys are investigated and compared with conventional biomedical alloys, that is titanium alloys and stainless steels. Corrosive parameters were determined using the potentiodynamic method. X-ray diffraction studies were performed to characterize the crystal structures. Microstructures of the prepared materials were examined using a scanning electron microscope, and surface hardness was measured by the Vickers method. The results show that investigated high-entropy alloys are characterized by simple structures. Three out of four tested high-entropy alloys had better corrosion properties than conventional implant alloys used in medicine. The Al_0.7_CoCrFeNi alloy was characterized by a corrosion potential of −224 mV and a corrosion current density of 0.9 μA/cm^2^; CoCrFeNiCu by −210 mV and 1.1 μA/cm^2^; TiAlFeCoNi by −435 mV and 4.6 μA/cm^2^; and Mn_0.5_TiCuAlCr by −253 mV and 1.3 μA/cm^2^, respectively. Therefore, the proposed high-entropy alloys can be considered as potential materials for biomedical applications, but this requires more studies to confirm their biocompatibility.

## 1. Introduction

High-entropy alloys (HEAs) are of great interest to researchers because they are a group of relatively new materials characterized by extraordinary properties [1,2,3]. They were first introduced in 2004 by Professor Yeh [4]. According to their definition, they contain between 5 and 13 components, and the content of each main component should be greater than 5 at.%. The entropy in HEA should be higher than 1.61R, where R is the gas constant. This entropy value results in a more frequent stabilization of simple solid solutions than in intermetallic phases [2]. Due to this, HEAs can exhibit remarkable mechanical and functional properties, such as excellent thermal stability, wear, and oxidation resistance [5,6,7,8,9]. This is related, among others, to the “four core effects” defined by Yeh [2]. These include the previously mentioned high-entropy effect, which contributes to the formation of simple solid solutions. The next is the sluggish diffusion effect, which causes, for example, the formation of nano-sized precipitates. The third effect is called the severe lattice-distortion effect and is caused by the differences in the atomic radii of the elements forming the alloy. It can cause a strengthening effect of an alloy; however, it may also affect the brittleness of the material. The last defined phenomenon, the cocktail effect, is related to the properties of HEA. It indicates that when multiple elements are mixed together, it can result in unexpected properties that single elements cannot achieve [10,11,12,13].

Combinations of different components forming high-entropy alloys provide great opportunities to obtain new materials for various applications. One area of modern materials research is the search for biocompatible metallic materials. Among all biomaterials, which include metals, polymers, composites and ceramics, metallic alloys have an important role due to their advantageous mechanical properties, mainly in the combination of high strength and sufficient ductility. However, the specificity of contact with human tissues creates high requirements for metal alloys, mainly in terms of their corrosion resistance. For this reason, three groups of alloys, such as austenitic steels, titanium alloys and cobalt alloys, are mainly used nowadays. Therefore, an entirely new alternative may be the application of high-entropy alloys in medicine. Some attempts have been made to examine selected high-entropy alloys for biomedical applications. One of the studied compositions is the TiZrNbTaMo alloy, which consists of two body-centered cubic (bcc) phases. The alloy, obtained by arc melting, is characterized by a Young’s modulus of 153 GPa, a compressive yield strength of 1390 MPa, and a surface microhardness of 4.9 GPa. Moreover, this alloy exhibits a much higher corrosion resistance in phosphate buffer solution than the conventional Ti6Al4V titanium alloy used for implants. It also has a much better pitting resistance than 316L stainless steel and CoCrMo alloys. The effect of varying titanium content in this composition was also investigated. Decreasing the titanium content results in an increase in surface microhardness and yield strength, and an improvement in wear resistance. It was shown that the properties of the Ti_0.5_CoCrFeNi alloy provided the best compromise between high compressive strength, plastic deformation, as well as corrosion and friction properties [14,15,16,17,18]. On the other hand, a TiZrHfNbTa alloy with a high biocompatibility and low magnetic susceptibility has also been designed. This alloy is reported to have a combination of a low Young’s modulus, high strength, and good ductility [19,20]. The literature also reports on alloy combinations such as FeMoTaTiZr [21], TiTaFeZrNb [22] proposed for use as metallic biomaterials.

Yeh et al. [4] studied the effect of aluminum addition on the transformation of crystal structure and the hardness of the CoCrFeNiCu alloy. Their results showed that it had a face-centered cubic (fcc) structure and a hardness of 133 HV. However, the introduction of aluminum resulted in a gradual change in microstructure from fcc to bcc, which affected the resultant hardness of the material. Nevertheless, it has to be stressed that we found no previous studies regarding the anti-corrosion properties of this alloy were. Another type of Al-containing alloy, TiAlFeCoNi, was studied by Edalati et al. It was found that this alloy exhibits a high hardness (above 600 HV) and a bcc structure. Moreover, studies showed that, compared to titanium alloys, it triggers a higher cellular metabolic activity of cells [23,24]. However, the Al_0.7_CoCrFeNi alloy and its properties are far better described in the current literature. It has a mixed fcc + bcc structure and exhibits a lamellar microstructure [25,26]. According to investigations, its hardness is about 350 HV [27]. No literature reports were found for the Mn_0.5_TiCuAlCr alloy.

As presented above, the Al-containing, high-entropy alloys, e.g., TiAlFeCoNi, as well as the CoCrFeNiCu alloy, have not yet been thoroughly examined. Most importantly, their corrosion resistance in a simulated body environment is still unknown. Therefore, the high-entropy Al_0.7_CoCrFeNi, CoCrFeNiCu and TiAlFeCoNi and Mn_0.5_TiCuAlCr alloys were selected for this study. The chosen method of preparation was induction melting. In this method, an induced current is used, which generates large amounts of heat in the metals, resulting in their melting. The advantage of this method is the stirring effect of the induction electromagnetic field, which allows a homogeneous cast to be obtained [28]. However, induction melting has limitations related to the maximum operating temperature of the crucible. Therefore, it was decided to create high-entropy alloys whose components have a melting point below 2000 °C. For comparison purposes, the same method was used to produce 316L steel and the Ti6Al4V titanium alloy, which are the metals of first choice in medical applications. The corrosion properties and hardness of the resulting conventional and high-entropy alloys were investigated and compared. Additionally, microstructure and XRD tests of the obtained HEAs were carried out.

## 2. Materials and Methods

### 2.1. Materials

Four high-entropy alloys were selected for the study: Al_0.7_CoCrFeNi, CoCrFeNiCu, TiAlFeCoNi, and Mn_0.5_TiCuAlCr. The choice of alloys was determined by a thorough literature analysis and the preliminary results of the authors’ own research. For comparison purposes, the conventional alloys used in biomedicine, that is 316L steel and Ti6Al4V, were also included. The compositions of the prepared alloys are shown in Table 1. 

### 2.2. Sample Preparation

High-entropy Al_0.7_CoCrFeNi, CoCrFeNiCu, TiAlFeCoNi, and Mn_0.5_TiCuAlCr, as well as conventional Ti6Al4V and 316L alloys were prepared by induction melting. While Ti6Al4V and 316L served as a reference, it was decided to prepare them using the same processing route as the high-entropy alloys, instead of utilizing them in their as-delivered state. This was carried out in order to eliminate the effect of the manufacturing technology selection on the obtained results.

Melting and casting was performed under vacuum and was preceded by purging a furnace chamber with argon. The purity of the raw materials was above 99.9%. The samples were remelted three times to achieve chemical homogeneity. Then, casts of the required dimensions and volume were obtained. From the obtained castings, samples for corrosion, surface hardness, X-ray diffraction (XRD) and microstructure were cut. For example, for the corrosion tests, ground and polished specimens of 8 mm diameter and 5 mm height were needed. On the other hand, samples for microstructure studies, after grinding, were additionally polished with a diamond suspension and etched with aqua regia.

### 2.3. Characterization Methods

The chemical composition and the microstructures of the obtained samples, obtained prior to the corrosion tests, were analyzed by a high-resolution scanning electron microscope (SEM-FIB DualBeam Scios 2, Thermo Scientific, Waltham, MA, USA). For imaging purposes, various detectors were used: the secondary electron (SE) Everhart–Thornley (ETD), as well as two in-lens detectors: SE, denoted as T2, and the backscattered electron (BSE) detector T1. This selection of the detectors was made in order to have the ability to present different features of the samples, such as their topography obtained after etching with aqua regia (SE); as well as to easily distinguish the phases obtained in the samples (BSE). Moreover, the chemical composition of the obtained phases was investigated with the use of an energy-dispersive X-ray spectroscopy (EDX) detector, which is an integral part of the used SEM-FIB microscope system (Ultra Dry EDS, Thermo Scientific, Waltham, MA, USA). In addition, hardness was measured by the Vickers method under a load of 98.07 N using a INNOVATEST hardness tester (Innovatest Europe BV, Maastricht, The Netherlands).

X-ray diffraction measurements at a room temperature were performed using an Empyrean Panalytical powder diffractometer (Malvern Panalytical, Malvern, UK) equipped with a Cu X-ray tube (*K*_α_ radiation, λ = 0.7093187 Å, 40 kV, 30 mA) and a PixCel1D strip detector. The scattered intensity was recorded by Bragg–Brentano geometry in a range of 2*θ* from 20° to 135°, in steps of 0.026261°. The standard used during X-ray refinements was LaB_6_ C660 crystallizing in a cubic system Pm-3m (space group no. 221). The phase analysis was carried out based on the Inorganic Crystal Structure Database (ICSD) using the HighScore program [29].

Corrosion resistance of the samples was investigated according to the requirements for metallic biomaterials. The corrosion stand utilized during the investigation is described in detail by Klekotka et al. [30]. A VoltaLab PGP 201 potentiostat and VoltaMaster 4 software were used for electrochemical tests. The experiments were carried out in a three-electrode system. A saturated calomel electrode of Hg/Hg2Cl2/Cl−REF421 (Radiometer Analytical, Loveland, CO, USA) was used as a reference electrode, while the counter electrode was an XM 140 platinum electrode (Radiometer Analytical, Loveland, CO, USA) with an area of 8 mm × 8 mm. Tests were carried out in 0.9% NaCl solution at room temperature in a measuring vessel made of borosilicate glass. The surface area of the samples exposed to the corrosive environment was 50.27 mm^2^. The electrochemical tests were followed by measuring the open circuit potential (OCP) to obtain a stable value. Potentiodynamic polarization measurements were performed at a scanning rate of 3 Mv·s^−1^. The test consisted of recording the anodic and cathodic polarization curves using polarization resistance methods and extrapolating the Tafel line to determine the values of corrosion potential and corrosion current density. The corrosion rate was calculated based on the methodology presented in the literature [31,32]. Moreover, for comparison purposes, a laser confocal microscope (Olympus Lext 4000, Olympus, Tokyo, Japan) was used to study the microstructure of the samples after the corrosion tests.

## 3. Results and Discussion

### 3.1. Microstructure

**Figure 1:**
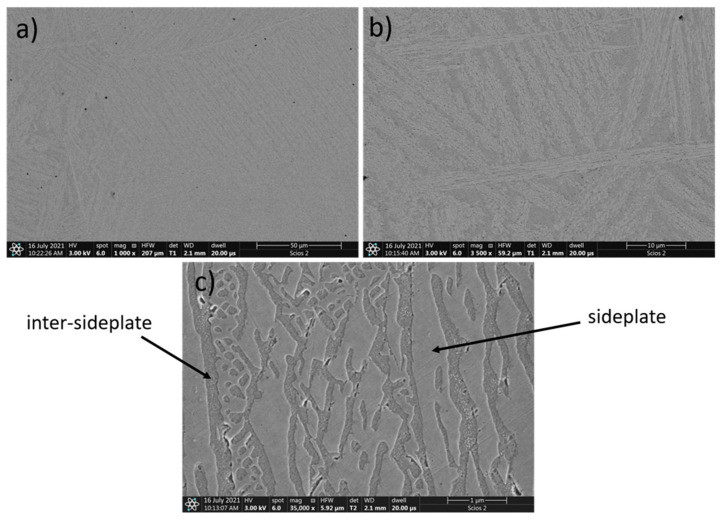
SEM images of Al_0.7_FeCrCoNi alloy, magnification: (**a**) 1000×, (**b**) 3500×, (**c**) 35,000×.

During the SEM observations, it was found that the chemical composition of the investigated HEAs greatly affected their resultant microstructure. In Figure 1, the microstructure of the high-entropy Al_0.7_CoCrFeNi alloy is presented. The results are supported by the EDS analysis of the chemical composition of the obtained alloy (Table 2).

In the Al_0.7_CoCrFeNi alloy, the occurrence of two phases is clearly visible, which corresponds well with the XRD results (Figure 1). The chemical composition indicates that the obtained phases vary in the aluminum content. The arrangement of these phases in the microstructure is complex (Figure 1). It is possible to observe bands, which are characteristic for the as-cast Widmanstatten structure, with the dominant α phase lower in aluminum (sideplates). Aluminum precipitated at the crystallization front, while the aluminum-rich β phase appeared in the spaces between the grains. Among bands, there is an equilibrium mixture of both phases (Figure 1).

**Figure 2:**
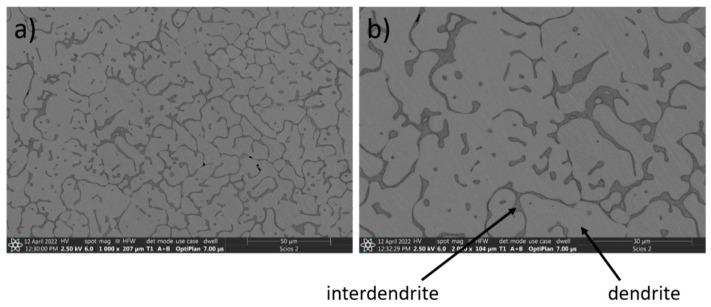
SEM images of CoCrFeNiCu alloy, magnification: (**a**) 1000×, (**b**) 2000×.

On the other hand, an entirely different microstructure was obtained for the CoCrFeNiCu high-entropy alloy (Figure 2). Its chemical composition is shown in Table 3. In this alloy, copper was used instead of aluminum.

In the microstructure of the CoCrFeNiCu high-entropy alloy (Figure 2), two phases can be identified. Copper has clearly segregated (Table 3). The bright dendrites, which are clearly seen in the BSE mode, are characterized by an even distribution of elements. However, compared with the other phase constituents, the Cu content in the dendrites is far lower than in the interdendrite areas (Table 3). The dark interdendritic phase (Figure 2) consists of mostly Cu, with small additions of other elements (Table 3).

**Figure 3:**
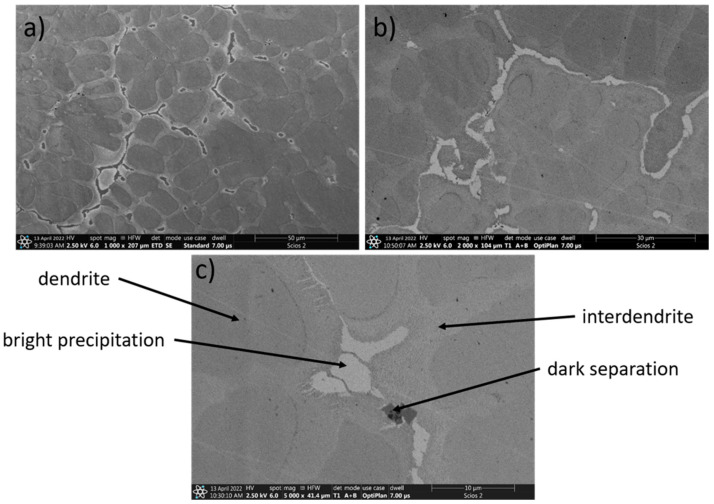
SEM images of TiAlFeCoNi alloy, magnification: (**a**) 1000×, (**b**) 2000×, (**c**) 5000×.

In Figure 3, the microstructure of the TiAlFeCoNi alloy is shown. A significant segregation of the alloying components in the obtained material can be observed, which leads to the formation of four separate phases (Figure 3c). However, the XRD signal from dark separations could be too weak due to the very small number of these precipitates when compared with the low-magnification microstructures (Figure 3a,b). In Table 4, the chemical composition of the obtained TiAlFeCoNi high-entropy alloy is presented.

As shown, compared with the other microconstituents, dendrites contain much less iron, while the number of other elements is similar. Moreover, the interdendritic areas have significantly less aluminum and more iron (Table 4). The dark separations consist mainly of titanium and aluminum, with small additions of other alloy components.

**Figure 4:**
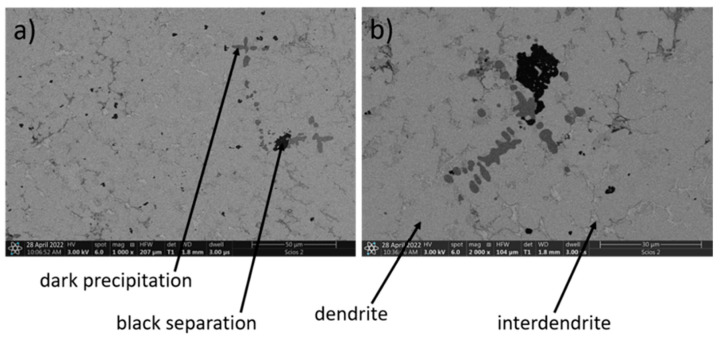
SEM images of Mn_0.5_TiCuAlCr alloy, magnification: (**a**) 1000×, (**b**) 2000×.

Figure 4 presents the microstructure of the high-entropy Mn_0.5_TiCuAlCr alloy. BSE SEM images of the Mn_0.5_TiCuAlCr alloy structure show a number of phases with significantly different chemical compositions, which indicates a high segregation of the elements. According to the data presented in Table 5, the microstructure of the Mn_0.5_TiCuAlCr alloy clearly exhibits dendrites that are rich in Ti, Cr and Al and have a reduced Mn and Cu content.

According to Table 5, the composition of interdendrites is similar to an intermetallic CuAl phase, with the addition of Mn. Furthermore, the composition of the black separation shows that it is probably based on the Al_3_Ti intermetallic phase, with the addition of other dissolved alloy components. The dark precipitation occurring in the alloy consists almost entirely of titanium, with trace amounts of other elements included.

### 3.2. Hardness

**Figure 5:**
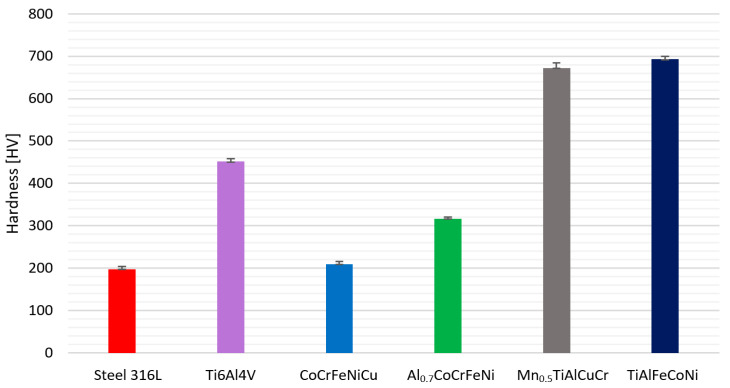
The average hardness of tested alloys.

Figure 5 shows the results of the Vickers hardness tests conducted for both the experimental high-entropy alloys, as well as the Ti6Al4V alloy and 316L steel reference samples.

The hardness of metallic biomaterials plays an important role, particularly when they are used in areas exposed to wear. The analyzed experimental high-entropy alloys differ significantly in hardness (Figure 5). It can be observed that the presence of titanium in the alloy causes a significant material strengthening effect. Out of all tested materials, Mn_0.5_TiAlCuCr and TiAlFeCoNi exhibit the highest hardness. Moreover, it has to be noted that their surface hardness is significantly greater when compared with the reference samples (316L and Ti6Al4V). In both Mn_0.5_TiAlCuCr and TiAlFeCoNi, AlTi-based precipitates occurred, which can also cause a precipitation strengthening effect. On the other hand, alloys without the Ti addition had a significantly lower hardness. However, out of all the experimental high-entropy alloys tested, the CoCrFeNiCu alloy is characterized by the lowest hardness. However, its hardness is similar to that of conventional 316L steel.

### 3.3. X-ray Diffraction Analysis

**Figure 6:**
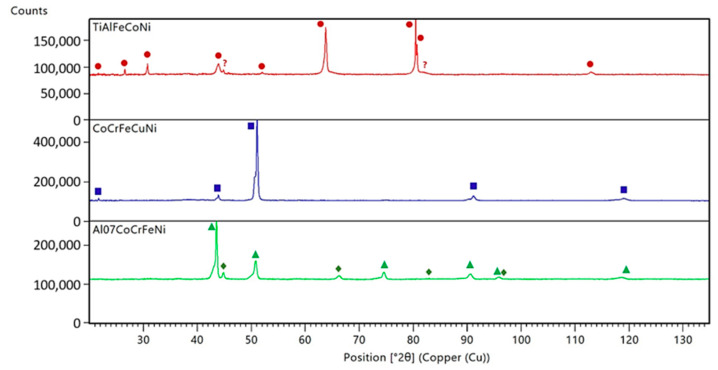
Room temperature X-ray diffraction patterns of TiAlFeCoNi—top red diagram, CoCrFeNiCu—middle blue diagram, and Al_0.7_CoCrFeNi—bottom green diagram. The symbols correspond to the different fcc phases (circles, squares, and triangles), while the bcc phase is represented by diamonds only.

The X-ray diffraction measurements allow the main phases occurring in the investigated samples to be identified (Table 6, Figure 6). The identified phases are marked on the X-ray diagrams according to a simplified system (see Table 6).

The results obtained for TiAlFeCoNi confirm the homogeneity of the system, although due to the limited number of diffraction reflections (Figure 6), they do not provide an unequivocal solution to the crystal structure. In fact, two options should be considered during the more sophisticated measurements. The former options leads to the single-phase primitive cubic structure with only two unrecognized impurities (question marks at the top of the red diagram). The second solution is in line with the bi-phase form of the TiAlFeCoNi sample, where the dominant ordered fcc phase is accompanied by an extra phase of the unknown, and not necessarily cubic symmetry. The single-phase CoCrFeNiCu alloy exhibits a disordered fcc structure, while the Al_0.7_CoCrFeNi sample discloses a predominant contribution of an ordered fcc phase and some amount of ordered bcc structure. The appropriate percentages of the phases’ volume contributions are equal to 96% and 4%, respectively. According to the XRD patterns shown in Figure 6 and taking into account the sensitivity threshold of the diffraction method itself, with a good approximation, the tested HEA alloys can be treated as homogeneous.

### 3.4. Corossion Properties

**Figure 7:**
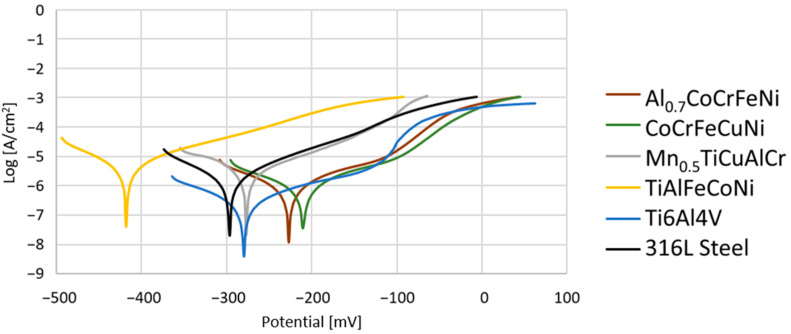
Potentiodynamic curves obtained for the investigated alloys.

Figure 7 presents the exemplary potentiodynamic curves obtained for all studied materials. The average values of the corrosion potential (E_corr_) and corrosion current density (i_corr_) obtained by the Tafel extrapolated method for individual groups are shown in Table 7. Additionally, the corrosion rate (v_cor_), and polarization resistance (R_p_) were determined for the tested samples.

Based on the obtained results, it has to be stressed that the inherited corrosion properties of the experimental high-entropy alloys were not worse than those of the conventional alloys used for tissue contact (Table 7). However, it has to be noted that the corrosion properties of the Ti6Al4V alloy and 316L steel differ from those reported in the literature. This mainly concerns the absence of a clear passivation range characterized by a stable value of the corrosion current density [33,34]. This phenomenon may be caused by the preparation method of the samples. Induction melting with further cooling in the air can cause a segregation of elements and the occurrence of casting defects, which significantly affect corrosion resistance. However, it should be noted that three out of four proposed high-entropy alloys obtained by the same manufacturing route showed better corrosion parameters than the conventional alloys (Figure 7, Table 7). The corrosion rate for the Al_0.7_CoCrFeNi alloy is the lowest and equals 11.7 μA/Year, and for the CoCrFeNiCu alloy, it is slightly higher (14.1 μA/Year). Nevertheless, the TiAlFeCoNi alloy has a significantly worse corrosion resistance and corrosion rate than other tested alloys (Figure 7, Table 7), which is confirmed by the microscopic observations of the alloy surface conducted after the corrosion tests.

**Figure 8:**
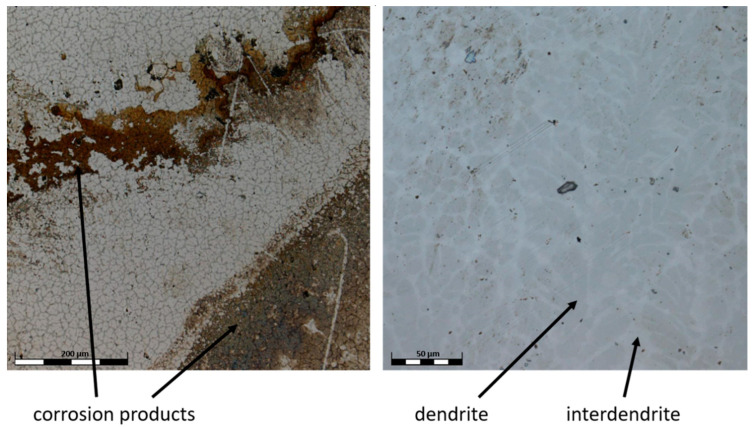
Confocal image of the TiAlFeCoNi surface after corrosion test.

**Figure 9:**
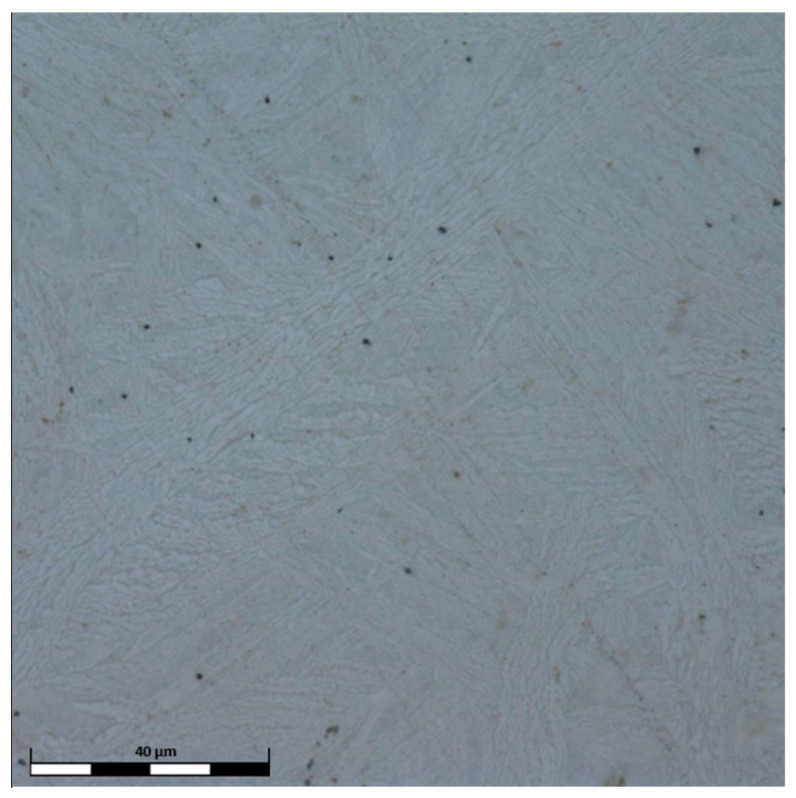
Confocal image of the Al_0.7_CoCrFeNi surface after corrosion test.

**Figure 10:**
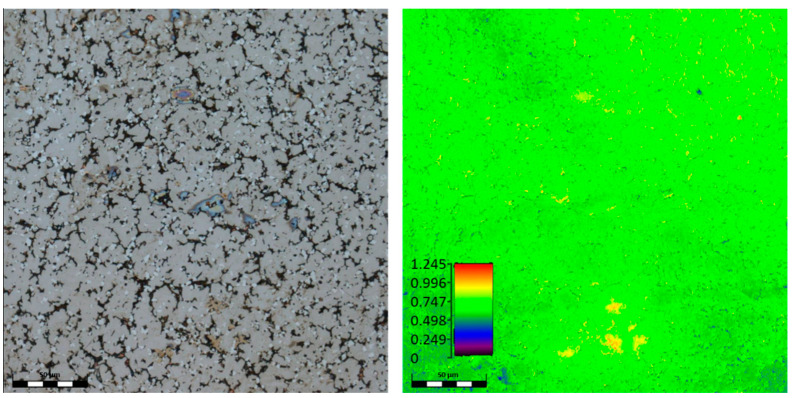
Confocal image of the Mn_0.5_TiCuAlCr surface after corrosion test.

**Figure 11:**
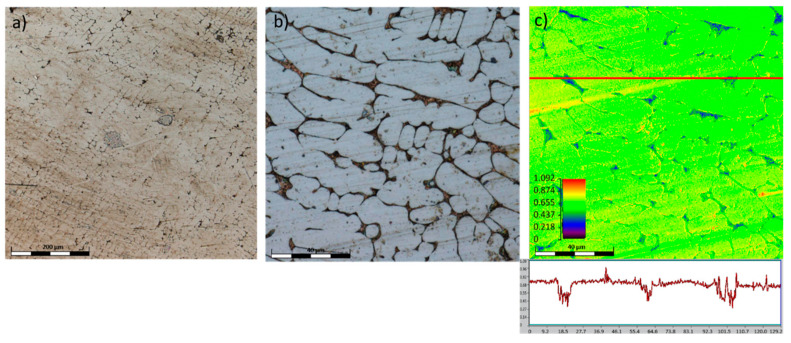
Confocal images of the CoCrFeNiCu surface obtained after the corrosion test: (**a**) low-magnification image, presenting the surface state of the sample, (**b**) high-magnification image, in which the corrosion stains in interdendritic areas are well discernible, (**c**) the color-scaled topography of image b; please note the depth of the periodically occurring corrosion pits.

**Figure 12:**
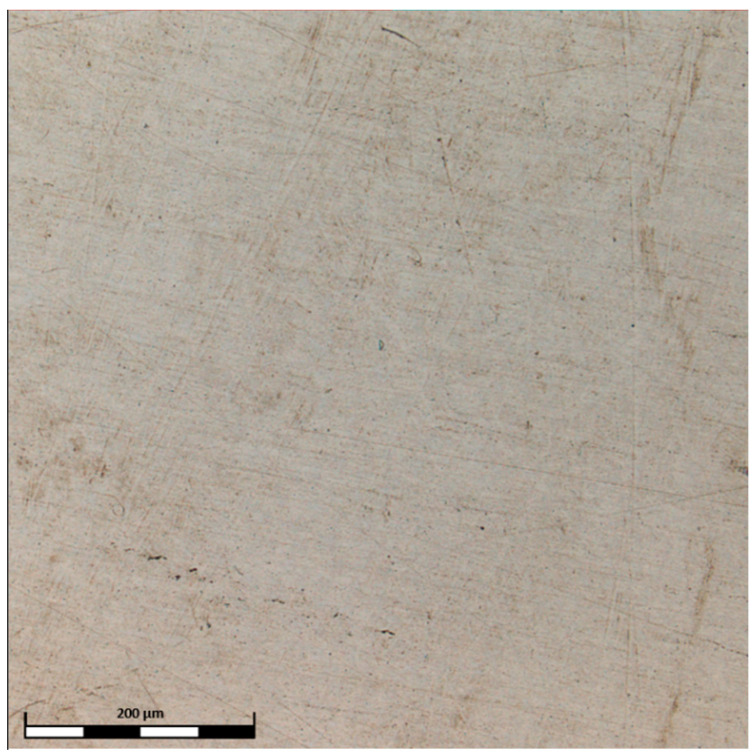
Confocal image of the 316L steel surface after corrosion test.

**Figure 13:**
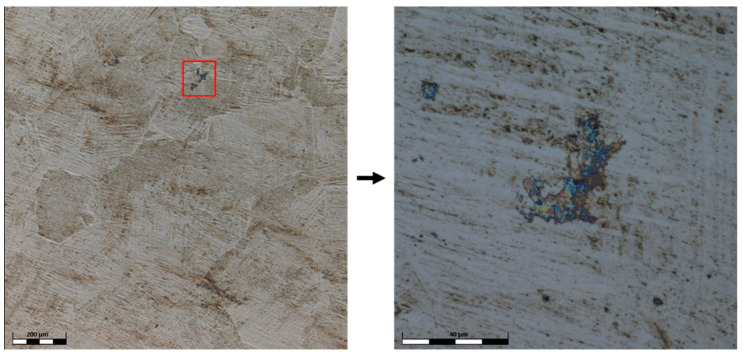
Confocal image of the Ti6Al4V surface after corrosion test.

The surface of the TiAlFeCoNi alloy shows large areas of discoloration, which are probably products of corrosion. There is also a highly visible structure through the etching of the interdendritic areas, which may indicate selective corrosion of the dendritic phase and interdendritic areas (Figure 8). Figure 9 shows that the corrosion test did not result in the appearance of pitting corrosion on the Al_0.7_FeCrCoNi alloy surface, but marks of general corrosion can be observed. However, in case of the Mn_0.5_TiCuAlCr alloy, local discoloration spots are noticeable, especially around precipitates in the interdendritic areas (Figure 10). Microscopic images of the CoCrFeNiCu alloy revealed that the indentations occur in the region of the interdendritic spaces (Figure 11). In comparison, after the corrosion test, no pitting or other signs of corrosion damage were observed on the surface of 316L steel (Figure 12). It can be concluded that this process has a general character. On the other hand, in the two-phase Ti6Al4V alloy, the structure also became clearly visible after the corrosion test. Grains of an α + β mixture and a local discoloration were noticeable. While pitting was observed, local discoloration was mainly seen in the area of casting defects (Figure 13). To conclude, based on the obtained corrosion tests results, three of the composed high-entropy alloys can be considered as potential biomaterials. However, further studies are needed in order to establish their mechanical and tribological properties, as well as to test whether they have a cytotoxic effect on human cells.

## 4. Conclusions

Based on the obtained results, the following conclusions can be drawn:

1. The studied high-entropy alloys crystallize mainly as solid solutions with simple crystal structures of high symmetry. Both TiAlFeCoNi and CoCrFeNiCu samples seem to be disordered single phases of an fcc type structure, but disclose the different types of space group. The neglectable contribution of two unrecognized peaks visible on the TiAlFeCoNi diagram does not influence the quality of refinements. The bi-phase Al_0.7_CoCrFeNi sample has the predominant contribution of an ordered fcc phase and a neglectable amount of ordered bcc structure.
2. Three out of four selected high-entropy alloys showed good corrosion properties. CoCrFeNiCu and Al_0.7_CoCrFeNi exhibited lower values for corrosion potentials and lower corrosion current density, as well as significantly lower corrosion rates, which may suggest a better corrosion resistance than the reference implant alloys (steel 316L and Ti6Al4V). These results indicate that these alloys can be considered as potential alloys for biomedical applications. This, of course, still requires many more studies to confirm their biocompatibility.
3. The presence of titanium in the high-entropy alloys (TiAlFeCoNi, Mn_0.5_TiCuAlCr) resulted in a significant strengthening effect on the materials, which could potentially result in good wear resistance. However, out of all samples tested, when compared with all investigated alloys, the TiAlFeCoNi alloy exhibited the worst corrosion properties, which excludes it as a material for biomedical applications. On the other hand, Mn_0.5_TiAlCuCr showed corrosion characteristics similar to Ti6Al4V, which combined with a high hardness, may determine its application as a friction element for biomedical implants. Nevertheless, in the long run, the precipitates present in these alloys may contribute to a decrease in their anti-corrosion properties.

## Figures and Tables

**Table 1:** SEM Energy-Dispersive X-ray Spectroscopy (EDS) chemical compositions (at.%) of investigated alloys.

Series No.	Alloy	Composition [at.%]									
		Al	Co	Cr	Fe	Ni	Ti	Mn	Cu	Mo	V
**1**	Al_0.7_CoCrFeNi	13.82	21.86	21.40	22.13	20.79	-	-	-	-	-
**2**	CoCrFeNiCu	-	20.66	19.83	19.98	19.40	-	-	20.14	-	-
**3**	TiAlFeCoNi	18.85	19.83	-	20.19	20.24	20.89	-	-	-	-
**4**	Mn_0.5_TiCuAlCr	21.56	-	21.38	-	-	24.38	11.16	21.53	-	-
**5**	Ti6Al4V	10.88	-	-	-	-	85.06	-	-	-	4.06
**6**	316L	-	-	18.99	68.28	11.52	-	-	-	1.22	-

**Table 2:** The chemical composition of Al_0.7_CoCrFeNi high-entropy alloy.

Alloy	Area	Element (at.%)				
		Al	Co	Cr	Fe	Ni
Al_0.7_CoCrFeNi	global	13.82	21.86	21.40	22.13	20.79
	sideplate	10.45	22.89	22.11	23.85	20.71
	inter-sideplate	16.25	19.75	23.28	21.17	19.55

**Table 3:** The chemical composition of CoCrFeNiCu high-entropy alloy.

Alloy	Area	Element (at.%)				
		Co	Cr	Fe	Ni	Cu
CoCrFeNiCu	global	20.66	19.83	19.98	19.40	20.14
	dendrite	22.94	22.26	22.63	22.43	9.75
	interdendrite	3.82	2.77	4.00	9.17	80.23

**Table 4:** The chemical composition of TiAlFeCoNi high-entropy alloy.

Alloy	Area	Element (at.%)				
		Ti	Al	Fe	Co	Ni
TiAlFeCoNi	global	20.89	18.85	20.19	19.83	20.24
	dendrite	21.62	23.89	10.74	22.19	21.56
	interdendrite	23.55	3.55	41.49	17.42	13.99
	dark separation	48.15	33.19	9.28	4.77	4.61

**Table 5:** The chemical composition of Mn_0.5_TiCuAlCr alloy.

Alloy	Area	Element (at.%)				
		Mn	Ti	Cu	Al	Cr
Mn_0.5_TiCuAlCr	global	11.16	24.38	21.53	21.56	21.38
	dendrite	20.66	30.13	12.40	20.66	24.60
	interdendrite	7.28	0.86	68.96	21.95	0.95
	dark precipitation	0.90	94.78	2.04	1.16	1.11
	black separation	1.49	4.15	5.33	87.07	1.97

**Table 6:** Results of the phase analysis performed using ICSD database, taking into account only the relevant cubic space groups, unit cell parameters, and percentage of phase volume contribution Vol.% obtained from XRD data.

Sample	Crystal Structure Space Group; Unit Cell Parameter [Å]	Vol. [%]	Order State; Graphic Symbol of the Phase
TiAlFeCoNi	Pm-3m (no.221); 5.8262 ± 0.0002	100	disordered fcc; red circle
	Fm-3m (no.225); 5.8271 ± 0.0008	100	ordered fcc; red question mark
CoCrFeNiCu	Pm-3m (no.221); 3.5745 ± 0.0001	100	disordered fcc; blue square
Al_0.7_CoCrFeNi	Fm-3m (no.225); 3.5948 ± 0.0001	94	ordered fcc; green triangle
	Im-3m (no.229); 2.8584 ± 0.0005	6	ordered bcc; olive diamond

**Table 7:** Selected corrosion parameters of the investigated materials.

Alloy	E_corr_ [mV]	i_corr_ [μA/cm^2^]	v_cor_ [μA/Year]	R_p_ [kΩ∙cm^2^]
Al_0.7_FeCrCoNi	−224	0.9	11.7	17.7
CoCrFeNiCu	−210	1.1	14.1	20.4
TiAlFeCoNi	−435	4.6	83.2	3.4
Mn_0.5_TiCuAlCr	−253	1.3	26.1	8.8
316L	−291	2.3	33.5	19.5
Ti6Al4V	−268	1	51.4	25.3

## Data Availability

The data presented in this study are available on request from the corresponding author.
